# Exertional heat stroke in sport and the military: epidemiology and mitigation

**DOI:** 10.1113/EP090686

**Published:** 2022-09-14

**Authors:** Julien D. Périard, David DeGroot, Ollie Jay

**Affiliations:** ^1^ Research Institute for Sport and Exercise University of Canberra Canberra Australia; ^2^ Army Heat Center Martin Army Community Hospital Fort Benning GA USA; ^3^ Thermal Ergonomics Laboratory Heat and Health Research Incubator Faculty of Medicine and Health University of Sydney Camperdown Australia

**Keywords:** exercise, heat illness, hyperthermia

## Abstract

**New Findings:**

- **What is the topic of this review?**
  Exertional heat stroke epidemiology in sport and military settings, along with common risk factors and strategies and policies designed to mitigate its occurrence.
- **What advances does it highlight?**
  Individual susceptibility to exertional heat stroke risk is dependent on the interaction of intrinsic and extrinsic factors. Heat policies in sport should assess environmental conditions, as well as the characteristics of the athlete, clothing/equipment worn and activity level of the sport. Exertional heat stroke risk reduction in the military should account for factors specific to training and personnel.

**Abstract:**

Exertional heat illness occurs along a continuum, developing from the relatively mild condition of muscle cramps, to heat exhaustion, and in some cases to the life‐threatening condition of heat stroke. The development of exertional heat stroke (EHS) is associated with an increase in core temperature stemming from inadequate heat dissipation to offset the rate of metabolically generated heat. Susceptibility to EHS is linked to the interaction of several factors including environmental conditions, individual characteristics, health conditions, medication and drug use, behavioural responses, and sport/organisational requirements. Two settings in which EHS is commonly observed are competitive sport and the military. In sport, the exact prevalence of EHS is unclear due to inconsistent exertional heat illness terminology, diagnostic criteria and data reporting. In contrast, exertional heat illness surveillance in the military is facilitated by standardised case definitions, a requirement to report all heat illness cases and a centralised medical record repository. To mitigate EHS risk, several strategies can be implemented by athletes and military personnel, including heat acclimation, ensuring adequate hydration, cold‐water immersion and mandated work‐to‐rest ratios. Organisations may also consider developing sport or military task‐specific heat stress policies that account for the evaporative heat loss requirement of participants, relative to the evaporative capacity of the environment. This review examines the epidemiology of EHS along with the strategies and policies designed to reduce its occurrence in sport and military settings. We highlight the nuances of identifying individuals at risk of EHS and summarise the benefits and shortcomings of various mitigation strategies.

## 1 INTRODUCTION

As homoeotherms, humans tightly regulate their internal body temperature. At rest in temperate conditions, a core temperature (*T*_core_) of ∼37°C is usually observed. Elevations in *T*_core_ due to exogenous and/or endogenous heat stress elicit thermoeffector responses (i.e., cutaneous vasodilatation and eccrine sweating) that in healthy individuals are usually sufficient to keep *T*_core_ within safe limits. However, if the thermal characteristics of the climate and/or clothing worn do not permit enough skin surface heat dissipation to offset the rate at which heat is internally generated, unchecked elevations in *T*_core_ occur (Cramer & Jay, 2016; Gagge & Nishi, 2011). While a 1–2°C rise in *T*_core_ can easily be tolerated by most people, a rise of 4–5°C can prove to be catastrophic for human health (Leon & Bouchama, 2015).

The mildest form of heat‐related illness is heat cramps, which are usually accompanied by heavy sweating, excessive thirst and fatigue (Leon & Kenefick, 2011; Maughan & Shirreffs, 2019). Sustained heat exposure, however, especially when coupled with vigorous exercise, can lead to the development of heat exhaustion, which is characterised by symptoms including dizziness, a weak rapid pulse, low blood pressure, nausea, vomiting, extreme fatigue and headache (Leon & Kenefick, 2011). The most extreme form of heat illness is exertional heat stroke (EHS), which is a life‐threatening condition and medical emergency characterised by central nervous system dysfunction, organ and tissue damage, and *T*_core_ frequently, but not always, in excess of 40°C (Bouchama et al., 2022; Laitano et al., 2019). For an in‐depth characterisation of the pathophysiology of exertional heat illness, the reader is referred to the following reviews: Bouchama et al. (2022), Laitano et al. (2019) and Leon & Bouchama (2015).

Two of the most common settings in which EHS is observed are competitive sport (i.e., elite and non‐elite) and the military. In elite sport settings, high levels of motivation and exertion can in some cases blunt sensory feedback from hyperthermia that under normal circumstances alter behaviour, and protective equipment and/or uniforms can potentially impede skin surface heat dissipation. In non‐elite sport settings, a wide range of physiological profiles related to factors such as age, fitness and health influence individual heat resilience, while the resources required for optimal heat protection may also be scarce. In military environments, heat illness risk is often exacerbated by heavy carrying loads, uniforms and protective equipment, and limited opportunities to self‐pace and thus down‐regulate metabolic heat production. Several strategies to mitigate the impact of environmental heat stress exist in both athletic and military settings, including heat acclimation, ensuring adequate hydration and mandated work‐to‐rest ratios (Army, 2022; McCubbin et al., 2020; Périard et al., 2021). A number of organisations have also published guidance for the prevention of heat‐related illness and EHS (Adams et al., 2021; Casa et al., 2015; Roberts et al., 2021) and a wide variety of risk factors have been identified (Bouchama et al., 2022; DeGroot et al., 2015; Leon & Kenefick, 2011). In this review, we examine the epidemiology of EHS, the most common risk factors, and policies designed to mitigate risk in sport and military settings. We aim to highlight the complexity in identifying those at risk of EHS across different forms of physical activity by outlining the nuances associated with its occurrence. We also aim to summarise the benefits, as well as the shortcomings, of different exertional heat illness mitigation strategies.

## 2 EXERTIONAL HEAT STROKE IN SPORT

**FIGURE 1:**
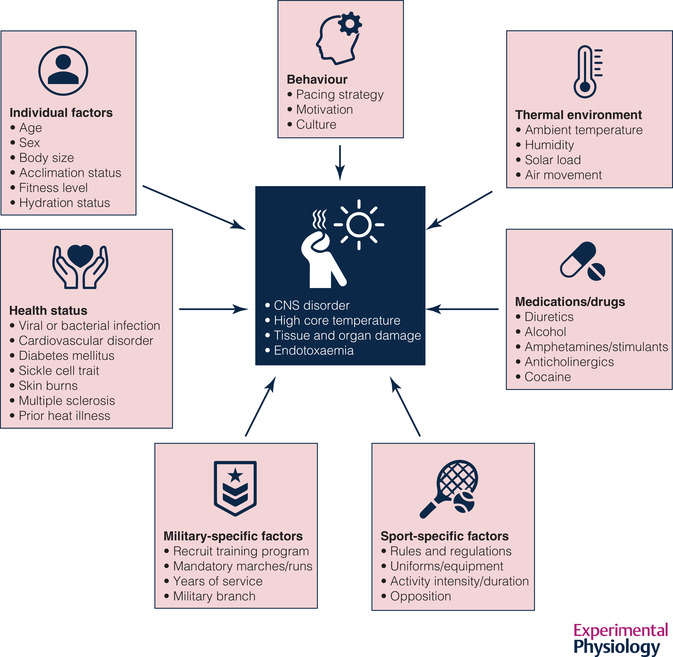
Factors associated with an increased risk of exertional heat stroke. Individual susceptibility to exertional heat stroke is dependent on the interaction of both intrinsic and extrinsic factors.

In athletes, individual susceptibility to EHS is dependent on several intrinsic and extrinsic factors, including environmental, individual and organisational factors, health conditions, medication and drug use, and behavioural responses (Figure 1) (Casa et al., 2015; Leon & Bouchama, 2015; Roberts et al., 2021; Westwood et al., 2020). In contrast to classic heat stroke, which affects mostly vulnerable populations (e.g., young children and older adults) exposed to environmental heat stress, EHS often occurs in healthy young individuals considered low risk and performing vigorous physical activity in hot or cool environmental conditions (Leon & Bouchama, 2015). EHS is the second most common cause of non‐traumatic death in competitive athletes (Maron et al., 2009). In a retrospective analysis of long‐distance running events in Tel Aviv from 2007 to 2013, it was reported that for every serious cardiac adverse event at races, 10 serious cases of EHS occurred (Yankelson et al., 2014). However, determining the exact prevalence of EHS across different sports is problematic (Howe & Boden, 2007), due to inconsistent exertional heat illness terminology, diagnostic criteria and data reporting (Bouchama et al., 2022; Gamage et al., 2020; Schwellnus et al., 2019).

Along with the prevailing environmental conditions, the prevalence of EHS is related to the demands of the sport (i.e., individual or team), such as exercise duration, intensity, clothing, and rules and regulations (e.g., rest and hydration breaks) (Casa et al., 2005, 2015; Hosokawa et al., 2021). In contrast to recreational activities (e.g., jogging) in which work rate is self‐regulated, physical activity patterns, and thus metabolic heat production, are often dictated by the event and/or opponent during competition (e.g., marathon or soccer match). In sports like American football, field hockey and lacrosse, players are required to wear different levels of protective equipment, such as helmets and shoulder pads. This equipment creates a microclimate above the skin surface that impairs heat dissipation, and its additional mass increases the rate of metabolic heat production (Armstrong et al., 2010; Montain et al., 1994). Accordingly, it has been shown that the risk of exertional heat illness is 11 times greater in American football players than in other high school sports (Kerr et al., 2013). American football has also been identified as the sport with the greatest number of EHS fatalities (Gamage et al., 2020; Grundstein et al., 2018; Yeargin et al., 2019). Between 1995 and 2019, 67 EHS deaths were recorded in the USA, 48 in high school students, 14 in college athletes, two in the professional ranks, two in organised youth sport and one in middle school (Kucera et al., 2020). Unfortunately, this is likely an underestimation of the number of EHS deaths, as reporting requirements can vary, if they exist at all. In addition to the clothing and protective equipment worn by players, the risk of EHS may be increased in linemen due to the static nature of position‐related activities and lower self‐generated air velocities, which reduce convective and evaporative heat loss (Deren et al., 2014). Excessive motivation and overexertion also appear to influence the rate of EHS in American college football, which is 2.6 times higher in competition than in practice (Yeargin et al., 2019). Although precise rates of EHS are unavailable, the incidence rate of heat illness in American football ranges from 0.6 to 41.9 per 10,000 athlete‐exposures, whereas it is much lower in other team sports like soccer (0.7 per 10,000 athlete‐exposures) and field hockey (0.8 per 10,000 athlete‐exposures) (Gamage et al., 2020). Conversely, the highest exertional heat illness incidence rates globally appear in endurance sports like running (674.0 per 10,000 athlete‐exposures) and cycling (401.1 per 10,000 athlete‐exposures) (Gamage et al., 2020).

In endurance running, the rate of EHS varies between races and seems to increase as distance decreases. From 2015 to 2019, the rate of EHS at the Boston Marathon was 3.7 per 10,000 athlete‐exposures (Breslow et al., 2021). A similar rate of exercise‐associated collapse with concomitant hyperthermia (3.0 per 10,000 athlete‐exposures) was noted by Roberts (2000) over a 12‐year period at the Twin Cities Marathon. In contrast, a much higher EHS incidence rate of 12.9 per 10,000 athlete‐exposures was reported over a 3‐year period at the Cincinnati Marathon, with the rate considerably higher in men (20.0 per 10,000 athlete‐exposures) than women (3.9 per 10,000 athlete‐exposures) (Divine et al., 2018). Others have reported incidence rates between 0.9 and 10.0 per 10,000 athlete‐exposures during half‐marathon races (Divine et al., 2018; Hawes et al., 2010; Schwabe et al., 2014; Sloan et al., 2015) and between 10.0 and 21.0 per 10,000 athlete‐exposures during 10‐km road races (Breslow et al., 2019; DeMartini et al., 2014; Demartini et al., 2015; Grundstein et al., 2019; Hosokawa et al., 2018). The higher rate of EHS incidence in shorter races stems from runners maintaining a faster running speed and rate of metabolic heat production, which accelerates the increase in *T*_core_ relative to longer races (Adams et al., 2016; DeMartini et al., 2014; Grundstein et al., 2019). In other athletes, excessive motivation (e.g., peer pressure, desire to win, prize money, qualifying spot) may lead to overexertion (Adams et al., 2016; Howe & Boden, 2007; Roberts, 2006) by sustaining a physical effort that is unmatched to fitness, a key risk factor for EHS (Rav‐Acha et al., 2004). Notwithstanding the variability in EHS prevalence, it has been demonstrated that the greatest proportion of those experiencing EHS are young (<30 years) fast runners of both sexes (Breslow et al., 2021). A linear correlation has also been demonstrated between EHS incidence and the severity of the thermal environment (usually quantified by wet‐bulb globe temperature: WBGT) (Breslow et al., 2021, 2019; DeMartini et al., 2014; Roberts, 2000; Sloan et al., 2015). These factors, along with the potential for dehydration to impair thermoregulatory heat loss mechanisms (Nadel et al., 1980; Sawka et al., 1985), may predispose athletes to hyperthermia and EHS, especially if they have experienced a recent illness and are immunocompromised (Leon & Bouchama, 2015).

In elite athletics, the incidence rate of EHS is quite low, with none reported during the Beijing 2015 (WBGT: 24–27°C) (Périard et al., 2017) and Doha 2019 (WBGT: 24–31°C) (Racinais et al., 2022) World Athletics Championships. Despite no cases of EHS, self‐reported history of heat illness symptoms in sprint, field and endurance athletes during the 2015 Championships indicated an EHS incidence rate of 163.0 per 10,000 athlete‐exposures (Périard et al., 2017). A ‘low number’ of EHS was reported in a study examining the incidence rate of exertional heat illness across seven World and European Championships between 2009 and 2018 in apparent temperatures ranging between 15°C and 27°C (Universal Thermal Climate Index: UTCI) (Hollander et al., 2021). The authors did not specify the number of EHS cases, as these were classified as heat‐related illnesses and evaluated collectively (i.e., heat syncope, heat exhaustion, hyperthermia and exertional heat injury). As with athletics, no EHS cases were reported during the 2016 Road Cycling World Championship in Qatar (∼27.1°C WBGT) (Racinais et al., 2020). Self‐reported history of EHS in a cohort of athletes competing in the championship indicated an incidence rate of 1159.4 per 10,000 athlete‐exposures. This self‐reported rate is extremely elevated, particularly since the incidence rate of exertional heat illness during the event was 31.0 per 10,000 (Racinais et al., 2020), even with 25% of participants in a separate study exceeding a *T*_core_ of 40°C and one reaching 41.5°C (Racinais et al., 2019). It is also much higher than the incidence rate of exertional heat illness in recreational cyclists across three studies (∼401.0 per 10,000 competitors) (Dannenberg et al., 1996; Friedman et al., 1998; Townes et al., 2005).

The incidence rate of EHS appears to be quite low in many other sports, including professional beach volleyball where no cases were reported over a 3‐year surveillance period (Bahr & Reeser, 2012). Similarly, no cases of EHS were reported in swimming, although concerns about hyperthermia due to water temperature were raised following the death of an elite swimmer during a 10‐km open water race (Macaluso et al., 2013). Despite several studies examining exertional heat illness in tennis (Sell et al., 2013; Smith et al., 2018a, 2018b), the only data available on EHS appear to be from a 6‐year injury surveillance study conducted during US National Boys Tennis Championships, which reported no EHS cases (Hutchinson et al., 1995). Similarly, despite one severe case of EHS occurring in rugby league football requiring repeated haemodialysis, assisted ventilation and supportive therapy more than 30 years ago (Savdie et al., 1991), along with the recent EHS‐related deaths of a teenager (Weber, 2018) and an adult professional (BBC, 2018), both during training, the incidence rate in rugby league football does not seem to have been determined (Gissane et al., 1998). These cases reinforce the multifactorial and diverse nature in which EHS can occur. In the first case, the player was experiencing a mild upper respiratory tract infection while playing in 24.1°C; in the second case, the player was training in 44°C; while in the third case, the player was undertaking a training run at a lower ambient temperature but a higher humidity. In the sport of triathlon, a 6‐year retrospective analysis of UK‐based races produced an EHS incidence rate of 1.74 per 10,000 athlete‐exposures. In a separate analysis of two races held in early‐ and mid‐summer in Australia, EHS was suspected in 3 of 15 participants (434.8 per 10,000 athlete‐exposures) during an early‐summer race (∼24°C WBGT), with no cases occurring during a mid‐summer event (∼25°C WBGT) (Gosling et al., 2008). This observation highlights the elevated risk of EHS during early season competitions when athletes have yet to heat acclimatise (Brown et al., 2022) and may not be prepared for unseasonably hot weather.

It is clear that the prevalence of EHS must be better characterised across several sports. While this characterisation is often complicated by inconsistent terminology, diagnostic criteria and reporting, a recent consensus statement by Schwellnus et al. (2019) has provided the framework for reporting medical encounters during community‐based endurance events, complementing that of specific sports (Fuller et al., 2006, 2007; Mountjoy et al., 2016; Timpka et al., 2014). The statement defines standard terms for describing injury and illness‐related medical encounters, along with their severity and timing, and diagnostic categories, including those related to exertional heat illness. Described are also uniform procedures for collecting and reporting data to allow for information from different sports to be aggregated and compared. Adopting such a framework is likely to provide more accurate estimates of EHS incidence rates and the predisposing factors, including individual factors (e.g., age and sex; Giersch et al., 2022), environmental conditions (e.g., temperature, humidity and air movement) and health status (e.g., injury and prior illness). It is also anticipated that more accurate incidence rates will enhance organisational heat illness preparedness and ultimately athlete safety.

## 3 EXERTIONAL HEAT STROKE RISK MITIGATION IN SPORT

**FIGURE 2:**
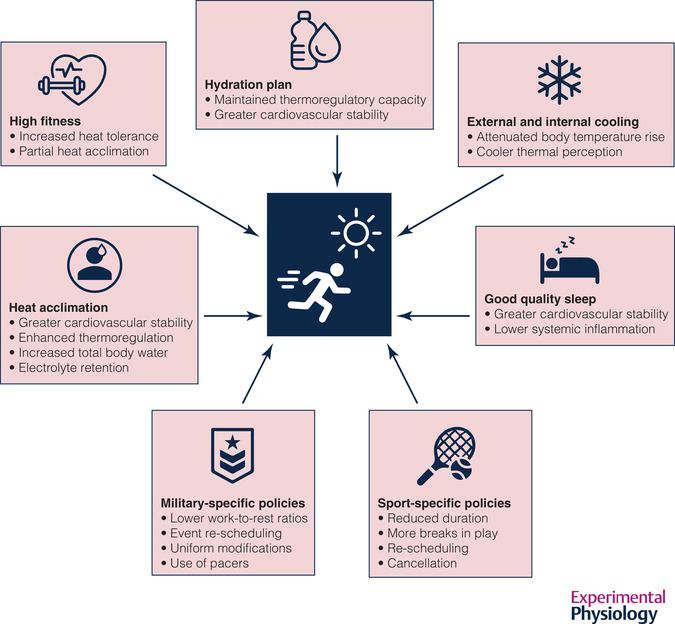
Strategies and policies designed to mitigate the risk of exertional heat stroke. Individual athletes and military personnel can attenuate the risk of experiencing exertional heat stroke by ensuring that they are adequately fit, acclimated/acclimatised to the environmental conditions and properly hydrated prior to and during vigorous exercise in the heat. Cooling strategies (external and internal) employed prior to and during exercise, along with good quality sleep, may also mitigate the risk of exertional heat stroke. Finally, empirically based sport and military‐specific heat policies are critical in safeguarding the health of athletes and military personnel.

Individual susceptibility to EHS is dependent on several factors, some of which are modifiable (e.g., heat acclimation, fitness, hydration status, sleep) (Pryor et al., 2020). As such, prevention and mitigation strategies addressing these factors can be employed to reduce the risk of EHS (Figure 2). Heat acclimation and acclimatisation, which refer to periods of repeated heat exposure in artificial (i.e., laboratory) and natural (i.e., outdoor) settings, respectively, lead to physiological and molecular adaptations that reduce the deleterious effects of heat stress and reduce exertional heat illness susceptibility (Périard et al., 2015; Sawka et al., 2011). These benefits are conferred via enhanced sweating and skin blood flow responses, plasma volume expansion, better fluid balance and cardiovascular stability, and acquired thermal tolerance (Horowitz, 2014; Périard et al., 2021). Fitness, or regular aerobic training in cool conditions, confers benefits similar to heat acclimation (e.g., lower resting *T*_core_, increased sweat rate) (Ravanelli et al., 2020; Sotiridis et al., 2020). This partial heat acclimation reduces physiological strain and increases exercise capacity in the heat (Alhadad et al., 2019). It is also recommended to ensure euhydration before exercise and replace a portion of sweat losses during exercise if possible, to mitigate the compounding effects of hyperthermia and dehydration (McCubbin et al., 2020; Périard et al., 2021). This is because severe dehydration impairs thermoregulatory function by reducing the sensitivity of thermoeffector responses to a given *T*_core_, which may exacerbate the development of thermal strain and increase EHS risk. Other mitigation strategies include pre‐ and per‐cooling (Bongers et al., 2017; Périard et al., 2021; Stevens et al., 2016), and ensuring adequate sleep (Pryor et al., 2020; Westwood et al., 2020).

Along with individual athlete strategies, event organisers can implement policies to modify or cancel events based on the prevailing ambient conditions and nature of particular sports (i.e., equipment and metabolic heat production) (Hosokawa et al., 2021; Racinais et al., 2015; Roberts et al., 2021). For the sake of simplicity, some sports utilise heat policies that employ a fixed air temperature cut‐off threshold for modifying or postponing play. While such an approach lends itself to widespread use given the availability of freely accessible online weather information, the level of physiological heat strain that can evolve at a fixed temperature is vast, with varying levels of humidity, wind and cloud cover substantially altering sweat evaporation and radiative heat load.

There are over 100 existing indices that offer a more sophisticated method for assessing the level of heat stress present in an environment than simply measuring air temperature (Havenith & Fiala, 2015). By far, the most frequently used approach in sport‐related settings is the WBGT index, which is widely advocated by organisations such as the American College of Sports Medicine (Roberts et al., 2021). Originally developed for managing heat stress risk of US military recruits during training activities (see Military section), advantages of the WBGT method include a single integrated value that fully or partially reflects all four environmental parameters that determine human heat stress risk. To be fully effective, though, all components of the WBGT (i.e., natural wet‐bulb temperature, black globe temperature and dry‐bulb temperature) must be physically measured at the site of play with correctly used and calibrated equipment, yet often WBGT values are estimated using only measures of temperature and humidity with assumptions applied about cloud cover and wind speed (Havenith & Fiala, 2015). Critical WBGT risk threshold ‘temperatures’ can be adjusted for the clothing, activity level and heat acclimation status of the athlete, making it usable across a wide range of sports. The WBGT should be interpreted with caution in environments with low air speeds, and with high temperature and low humidity where the limit to human heat dissipation is not determined by the climate, but by the ability to secrete sweat, as EHS risk may be underestimated (Budd, 2008).

In Australia, a country that contends annually with bouts of extreme heat throughout the sporting summer, several organisations have undertaken work to develop customised sport‐specific heat policies. Since the early 2000s, the National Rugby League (NRL) have utilised the Belding and Hatch (1955) Heat Stress Index, which evaluates the evaporative capacity of the environment (*E*_max_) relative to the evaporative heat loss requirements of the athlete (*E*_req_). The underlying principle is that the more *E*_req_ exceeds *E*_max_, the faster the rate of rise of *T*_core_ and thus EHS risk. In advance of the 2017 Rugby League World Cup, which hosted games in Port Moresby (Papua New Guinea), Cairns and Darwin (Australia) in October and November – all of which presented oppressively hot–humid conditions – the NRL heat policy was updated to account for the thermal characteristics of the clothing/equipment worn, the activity levels of different playing positions and the physiological/morphological characteristics of the athletes. In‐play cooling interventions and breaks in play based on their efficacy for reducing the rise in *T*_core_ during simulated play in climate chamber experiments were also integrated into the revised policy (Graham et al., 2021). In 2018, Cricket Australia developed its first extreme heat policy utilising a cricket‐specific 10‐point ‘Heat Stress Risk Index’ (HSRI) (Jay et al., 2019). The index scales heat exposure throughout the course of a full‐day cricket match based on a combination of hyperthermia risk, determined by assessing the ratio of *E*_req_ relative to *E*_max_, again accounting for sport‐specific equipment/clothing and activity levels, along with the rate of dehydration based on estimated sweat rates and an assumed rate of fluid intake. Upon reaching pre‐determined HSRI thresholds, first more frequent drink breaks are implemented, and then the duration of these drink breaks is extended. If a maximum HSRI value of 10 is reached, play can be suspended following consultation between medical and match officials, and if necessary, the Head of Cricket Operations.

## 4 EXERTIONAL HEAT STROKE IN THE MILITARY

In the US military, surveillance of exertional heat illness is facilitated by standardised case definitions, a universal healthcare system that links electronic medical records into a central repository, and a requirement to report all heat illness cases to service‐specific public health agencies. Comparison of incidence rates between athletic populations and the military is often difficult, if not impossible. While much of the sports literature uses athlete‐exposures as the denominator, the US military uses person‐years, requiring us to consider the epidemiology of exertional heat illness in the military separately.

The US Armed Forces Health Surveillance Division (AFHSD) publishes an annual report of heat illness incidence rates, broken down by diagnosis, gender, age, race, service and military status (Williams & Oh, 2022). In this and prior annual updates, statistical comparison of rates between sub‐groups was not conducted. The overall incidence rate for EHS was 0.37 per 1000 person‐years, with men having marginally higher rates than women (0.40 vs. 0.23 per 1000 person‐years). This is not a consistent finding in the literature, however, as others have reported that women are at greater risk of EHS than men (Carter et al., 2005; Nelson et al., 2018). Recruits had higher incidence rates than other military enlistees or officers, as did those aged 20 or younger (who are often recruits). The Marine Corps (0.72 per 1000 person‐years) and Army (0.67 per 1000 person‐years) had markedly higher incidence rates compared to the Air Force (0.08 per 1000 person‐years) and Navy (0.04 per 1000 person‐years) (Williams & Oh, 2022). Other reports have identified poor physical fitness, high body mass index (BMI) and initial entry training as risk factors (Bedno et al., 2014; Gardner et al., 1996; Nelson et al., 2018; Wallace et al., 2006). Underscoring the challenge of identifying risk factors associated with EHS (Figure 1), Stacey et al. (2015) found that traditional risk factors were entirely absent from almost half the cases in their report.

There are limited data on the long‐term sequelae of EHS. While elevated cardiac troponin has been linked to EHS severity in a rodent model (Audet et al., 2015), the long‐term effects on the myocardium are not clear. A retrospective review of military service members who suffered an EHS event earlier in life showed an increased death due to cardiovascular disease and ischaemic heart disease (Wallace et al., 2007). Similarly, a study of civilians with prior exertional heat illness history reported a two‐fold higher prevalence of acute ischaemic stroke, acute myocardial infarction and an almost three‐fold higher prevalence of chronic kidney disease (Wang et al., 2019).

## 5 EXERTIONAL HEAT STROKE RISK MITIGATION IN THE MILITARY

Whether in pre‐season American football, half‐marathon running, military training or other activities, there may be certain circumstances in which exertional heat illness can be expected to occur. History has taught that the 5‐mile run on day 1 and the 12‐mile foot march on day 4 of US Army Ranger School training is associated with a high risk of EHS (DeGroot et al., 2015). Due in part to unwavering standards for the Ranger School run and foot march events, coupled with individual motivation to excel, these events continue to produce a disproportionately high number of heat illness casualties during the summer months. However, an individualised risk assessment process, which helps to identify those at highest risk, has yet to be developed and validated. Such a process would allow event organisers and medical personnel to identify those individuals for targeted observation, or perhaps even restrict certain individuals from training or competing.

A factor that may be unappreciated, but has been known for many years, is individual motivation to excel. Recent data from the US Army demonstrates that 80% of all EHS casualties at Fort Benning, Georgia in 2021 occurred during foot march or run events (DeGroot, Henderson et al., 2022). These events are often conducted as a requirement to earn a credential such as Airborne School ‘Jump Wings’, or as a requirement to complete initial entry ‘basic’ training, thus imposing an extrinsic motivational factor on the individual. In a review of EHS deaths during military training, it was observed that ‘the tragedy of heatstroke is that it so frequently strikes highly motivated young individuals, under the discipline of work, military training and sporting endeavour. Under other circumstances, those same individuals would have rested when tired, drank when thirsty or remained home when ill’ (Shibolet et al., 1967). While preventive measures may be applied, it may be difficult, if not impossible, to eliminate the threat of EHS due to the uncontrollable factor of individual motivation.

A variety of heat stress mitigation efforts are routinely employed in the US Armed Forces (Figure 2). The WBGT index was originally developed at Marine Corps Recruit Depot Parris Island in the 1950s (Yaglou & Minard, 1957). While work‐to‐rest ratios and water consumption guidelines associated with the WBGT index have been revised over the years, the use of ‘heat categories’ to guide decision‐making remains (Army, 2022). Other administration controls include scheduling high‐intensity events during the coolest time of day, modifying the uniform worn to improve evaporative heat loss and limiting the weight of the load carried to reduce metabolic heat production. Several practices are routinely employed that lack scientific validity. For example, once a designated heat category is reached, uniform pants are un‐bloused from boots, coat sleeves are un‐cuffed and rolled up and troops in formation are placed at ‘double‐arm interval’, which is double the distance between troops. Each of these has the objective of improving airflow around the individual, but the effects on heat balance have not been quantified. One method that may have some validity is that of using a ‘pacer’ to mitigate risk during timed run and foot march events. Using Global Positioning System (GPS) feedback, an individual is placed at the front of the group and maintains the maximum acceptable pace; no individual is permitted to move at a faster rate than the pacer. Similarly, an individual may be placed at the rear of the group and maintains the minimum acceptable pace necessary to pass the event. Any individual who cannot maintain the minimum pace may be pulled off the course and not permitted to finish, with the assumption that if they were permitted to continue, they would attempt to sprint towards the end of the course, to meet the standard, only to collapse with an exertional heat exhaustion. Preliminary data from US Army Ranger School suggest that this minimum pace technique has merit, but further research is necessary (DeGroot, unpublished observations). In recent years, the practice of immersing the hands and forearms in cold water during rest breaks has gained acceptance in the US Army and has been incorporated into Army policy (Army, 2022; DeGroot, O'Connor et al., 2022). Immersion cooling takes advantage of the higher heat transfer coefficient of water relative to air and the large surface area‐to‐mass ratio of the hands and forearms to provide active cooling, particularly when the ambient water vapour pressure is high and evaporative heat loss is limited. Data from US Army Ranger School indicate that while overall exertional heat illness incidence was unchanged when immersion cooling was available, there was a shift in severity, with more heat exhaustion and fewer EHS casualties (DeGroot et al., 2015).

## 6 SUMMARY

The interaction of risk factors on physiological responses during exercise under heat stress increases the difficulty in identifying individuals at risk of EHS in both sport and military settings. While inconsistencies in exertional heat illness terminology and reporting hinder the determination of precise EHS incidence rates in sport, American football appears to be the sport in which EHS‐related death occurs with the highest prevalence. The rate of EHS incidence is particularly elevated in endurance sports, especially running, with the risk of EHS increasing during shorter races (e.g., 10 km) as athletes exercise at higher intensities than in longer races like the marathon. In many sports, including cycling, soccer, tennis, rugby league and open water swimming, additional EHS reporting is required to more accurately understand its prevalence and the approaches required to reduce its occurrence. The formulation of risk reduction plans in different sports, based on an understanding of EHS risk factors, may include strategies adopted by athletes (e.g., heat acclimation, hydration, monitoring health status) and event organisers (e.g., event modification policies, educational material), including the development of sport‐specific heat stress policies that account for the evaporative heat loss requirement of athletes and the evaporative capacity of the environment (i.e., ratio of *E*_req_ to *E*_max_). In the military, while traditional risk factors play a part in EHS risk, factors specific to the armed forces (e.g., recruit training, mandatory marches/runs, military branch) form an important part of this risk. The motivation to excel in tasks with high standards of achievement represents a setting in which disproportionally elevated exertional heat illness casualties occur. Notwithstanding, some forms of military training are adapted under thermally challenging environments to help reduce the incidence of heat illness and EHS. As with the military, adopting a standardised framework for collecting and reporting exertional heat illness data in sport will increase our understanding of the intrinsic and extrinsic risk factors associated with EHS, and guide the implementation of preventive strategies. Ultimately, the development of EHS is preventable and an understanding of the interaction between risk factors and mitigation strategies by athletes and event organisers, as well as military personnel, can reduce its prevalence.

## AUTHOR CONTRIBUTIONS

Julien D. Periard, David W. DeGroot and Ollie Jay developed the aim and scope of the review, drafted and reviewed the sections. All authors have read and approved the final version of this manuscript and agree to be accountable for all aspects of the work in ensuring that questions related to the accuracy or integrity of any part of the work are appropriately investigated and resolved. All persons designated as authors qualify for authorship, and all those who qualify for authorship are listed.

## COMPETING INTERESTS

None to declare.
